# The Effect of Incorporating Silica Stone Waste on the Mechanical Properties of Sustainable Concretes

**DOI:** 10.3390/ma13173832

**Published:** 2020-08-30

**Authors:** Saeid Abbasi, Mohammad Hemen Jannaty, Rabar H. Faraj, Shahriar Shahbazpanahi, Amir Mosavi

**Affiliations:** 1 Department of Civil Engineering, Sanandaj Branch, Islamic Azad University, Kurdistan 66169, Iran; saeed_abbasi_s@yahoo.com (S.A.); hemenjannaty@yahoo.com (M.H.J.); sh.shahbazpanahi@iausdj.ac.ir (S.S.); 2 Civil Engineering Department, University of Halabja, Halabja 46006, Kurdistan Region, Iraq; rabar.faraj@uoh.edu.iq; 3 Faculty of Civil Engineering, Technische Universität Dresden, 01069 Dresden, Germany; 4 Institute of Research and Development, Duy Tan University, Da Nang 550000, Vietnam; 5 Thuringian Institute of Sustainability and Climate Protection, 07743 Jena, Germany

**Keywords:** silica stone, sustainable concrete, sustainable construction, microstructure, compressive strength, sustainable development, materials cycle, green buildings, circular economy, sustainable materials, life-cycle assessment (LCA), sustainable development goals (SDGs), eco-friendly, recycled materials, waste management, building materials

## Abstract

Incorporating various industrial waste materials into concrete has recently gained attention for sustainable construction. This paper, for the first time, studies the effects of silica stone waste (SSW) powder on concrete. The cement of concrete was replaced with 5, 10, 15, and 20% of the SSW powder. The mechanical properties of concrete, such as compressive and tensile strength, were studied. Furthermore, the microstructure of concrete was studied by scanning electron microscopy (SEM), energy-dispersive X-ray spectroscopy analysis (EDX), Fourier transformed infrared spectroscopy (FTIR), and X-Ray diffraction (XRD) tests. Compressive and tensile strength of samples with 5% SSW powder was improved up to 18.8% and 10.46%, respectively. As can be observed in the SEM images, a reduced number of pores and higher density in the matrix can explain the better compressive strength of samples with 5% SSW powder.

## 1. Introduction

The construction sector is in need of alternate and replacement materials for cement due to its costly and pollution-inclined production system. Approximately 7% of total CO_2_ emissions are due to cement manufacturing [1]. In addition, nearly 650–920 kg CO_2_ is emitted during the production of one ton of cement [2]. Thus, recycled and alternative materials that can reduce cement consumption and with the possibility of eliminating CO_2_ emissions are of utmost importance [3].

Silica stone, which is used in various industrial applications, is one of the most important minerals in the Earth’s crust. Silica is used in the casting of factories, the supply of washing machine powder and cleaners, the process of sand making, the preparation of various types of chemicals, and finally, as the primary material for making glass and crystals [1]. Silica stone is crushed in stone crushing workshops to use in industry, and the remaining material in the pan after the screen is waste material. Disposal of these waste materials is a problem. Furthermore, the problem increases when these waste materials cannot be biodegradable [2]. The average size of SSW powder is 1–30 micron. The purity of silica stone waste, SSW powder is 96% in industrial-grade. The disposal of SSW powder in landfills causes contaminants. To avoid environmental pollution, SSW powder might contribute as a replacement of cement. Therefore, the use of this waste material in cement is important in concrete production not only for economic reasons [3] but also for technical ones [4]. Some investigations have been carried out to investigate the application of silica stone as aggregate [5,6]. Furthermore, many investigations have been done to study the effect of silica on concrete [7], waste glass [8,9], and glass powder [10,11]. For instance, Serifou et al. reported that waste glass can reduce the compressive strengths of samples due to the cracking potential of glass when used as coarse aggregate [12]. Yu et al. [13] investigated the strength of lightweight concrete containing lightweight waste glass as fine aggregate. Furthermore, many studies of silica derivatives in concrete such as silica fume [14] and nano-silica [15,16,17] have been presented. They observed that the additive of nano-silica as a modifying agent increased the compressive strength of samples [18,19].

All research has reported that silica derivatives not only reduce cement consumption but enhance the qualities of concrete, such as mechanical properties and microstructures. However, little research has been carried out to study the effect of silica stone waste (SSW) on mechanical properties and the microstructure of concrete. The SSW can be an appropriate sustainable unusual material as a replacement of cement while increasing the compressive strength [20]. The compressive and tensile strength of concrete samples with 5, 10, 15, and 20% SSW powder at 7, 28, and 90 days were tested to investigate the use of SSW powder as a feasible alternative material to cement. SEM, EDX, FTIR, and XRD tests were presented to identify the chemical and microstructure component.

## 2. Materials and Methods

**Figure 1:**
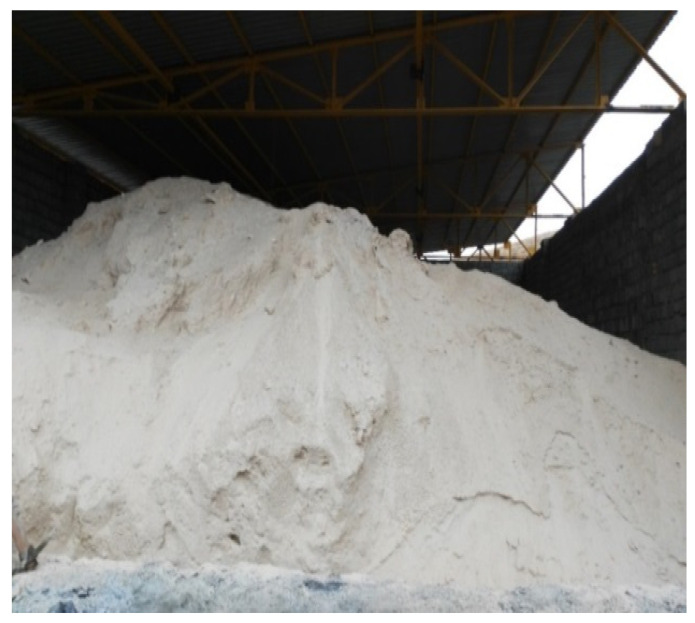
Silica stone waste (SSW) powder depot before the sieve.

**Figure 2:**
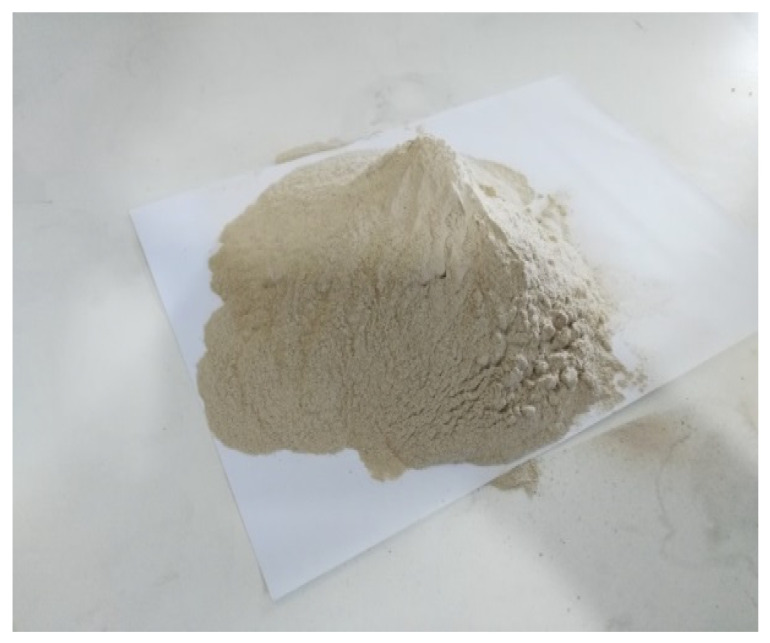
SSW powder after sieve.

In this study, type I Portland cement produced in Kurdistan with grade 30 (provided by Kordestan Cement Co, Bijar, Iran), following the ASTM C150 A [21], was utilized. This cement had a specific gravity of 3.00 g/cm^3^ with a time setting equal to 120 min. The Blaine fineness was 2880 cm^2^/g. The SSW powder was provided by Jahan Sang Company located in Azandaryan, Hamedan, Iran. The SSW powder before sieve used in this study is shown in Figure 1. SSW was filtered through a 10 μm sieve in order to remove any external materials, (Figure 2).

**Figure 3:**
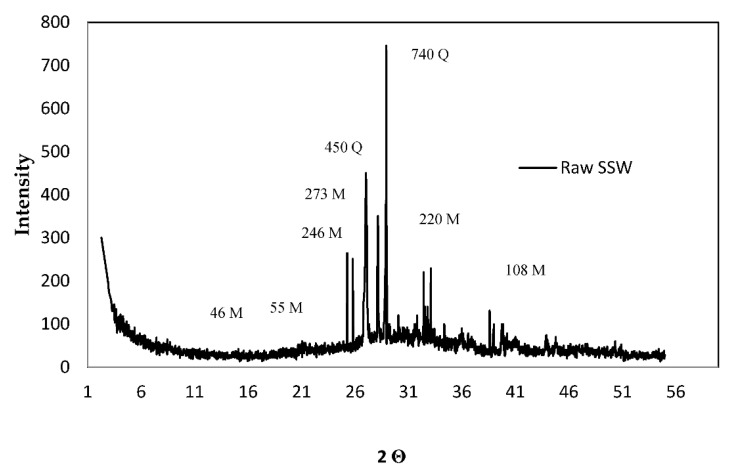
The X-ray diffraction graph of raw SSW powder.

Table 1 shows the chemical and physical components of cement and SSW powder by X-ray spectrometry (XRF) (tested at the University of Kurdestan, using a SHIMADZU 3001 facility, made in Japan). The chemical components of cement were provided by the manufacturer. An X-ray spectrometry test was performed to trace chemical components of the SSW powder. The major chemical component of SSW was SiO_2_ with 96.24%. The laser particle size analyzer test (measured at the University of Kurdestan, using a TSCAN test facility made in Czech Republic) was carried out to evaluate the average size of the SSW powder. The average size of the SSW powder was 8 microns with a density of 4.1 g/cm^3^. The X-ray diffraction (XRD) analysis of raw SSW (measured at the University of Kurdestan, using a TSCAN test facility made in Czech Republic) powder is shown in Figure 3. As shown in Figure 3, major amorphous (SiO_2_) and quartz (SiO_2_) phase were traced [22,23]. The highest peak location was identified in the XRD pattern from 20° to 40° (2 Theta) which was reported by previous efforts [24,25].

**Figure 4:**
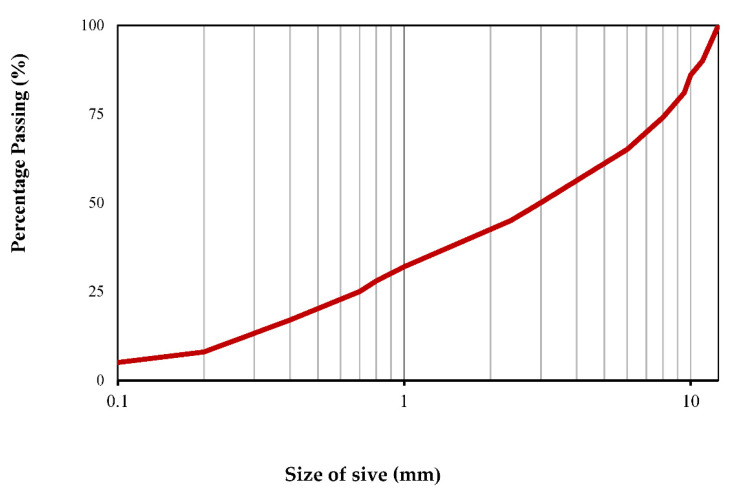
Grading curves of mixed aggregate.

Tap water with pH = 7 was used in mixing water according to ASTM C128 [26] for preparing concrete. To examine the effect of SSW powder on the test, the water to binder ratio was kept constant at 0.41. The aggregates were washed, dried, sieved, and graded, according to ASTM C128 [23]. The aggregates used were crushed granite stone, which according to ASTM C128 [26] is categorized as coarse aggregate with 2.6 density and maximum particle sizes of 12 mm and natural river sand as fine aggregate with 2.45 density and maximum particle sizes of 4.75 mm. The grading curve used for concrete is given in Figure 4. Aggregate grading was kept constant in all mix designs. Aggregates were kept in SSD situations when used in the mixture to prevent the effect of aggregate on free water content of mixtures [27].

### Mix Design of Concrete and Test Process

The mix designs of concrete were shown in Table 2. Cement was replaced by 5, 10, 15, and 20% SSW powder. In this paper, the dry mix method was used to prepare concrete. I n a drum mixer, SSD conditions coarse and fine aggregate were blended for 120 s and after that the cement was added and continued to mix for another 120 s. Next, the SSW powder was added and blended for 120 s. This was followed by slowly adding water and continuing to mix for 180 s. After stopping the drum, the mixer was covered with wet burlap and the concrete placed in molds ASTM C511 [28]. After one day of casting, molds were opened and put in water until 7, 28 and 90 days. The temperature at the test site was 29 °C.

After measuring the slump of fresh concrete, one hundred and fifty samples with standard cylinders were cast. To reduce error, five samples were made at each age of at 7, 28, and 90 days and the averages of these five sample results were used to determine the strength of samples. Seventy-five of those specimens were made to calculate the compressive and seventy-five specimens for tensile strength as control samples for all ages.

## 3. Results and Discussion

### 3.1. Concrete Slump

**Figure 5:**
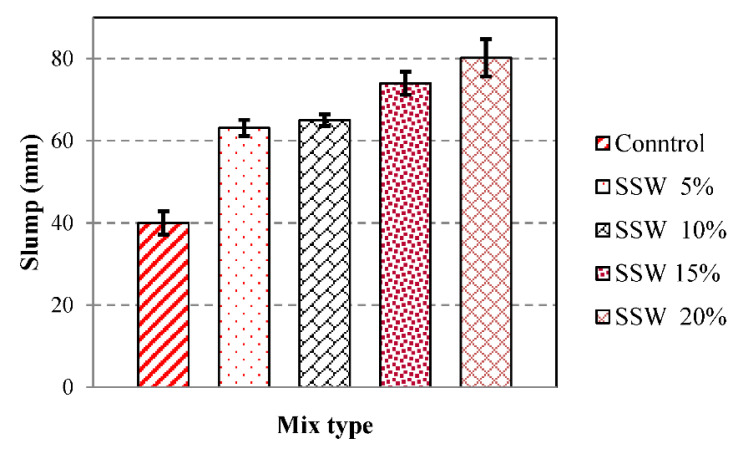
Slump of fresh concrete samples.

The slump of fresh concrete is conducted to evaluate the workability and pumping ability [29]. Figure 5 shows the slump of fresh concrete. The control slump sample was 40.0 mm and by adding 5, 10, and 15% SSW to the concrete, the slump of specimens increased to 52.5, 65.0, and 74.0, respectively. Figure 5 shows that adding a greater amount of SSW powder, 20%. increased the slump to 80.2 mm. The findings demonstrate that the slump of samples containing the SSW powder increase could be due to decreased water demand, which cannot be absorbed by SSW powder. Furthermore, the SSW powder might fill in the holes and consequently increase the slump.

### 3.2. Compressive Strength of Samples

**Figure 6:**
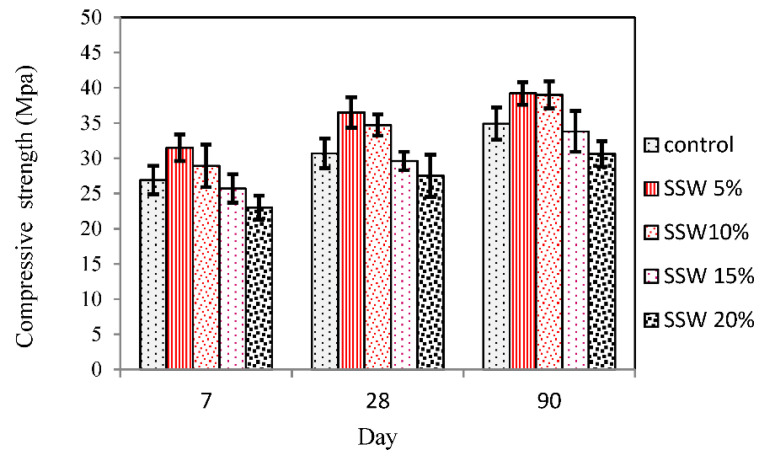
Effect of SSW on compressive strength of samples.

The results of the compressive strength of samples according to ASTM C39 [29] are shown in Figure 6. When 5% SSW was added to the sample, the compressive strength rose compared to the control samples by 17.1%, 18.8%, 12% at 7, 28, and 90 days, respectively. The high amount of SiO_2_ in SSW powder might be attributed to the improvement of strength and rise of the rate of C-S-H due to the cement consumption. Furthermore, SSW powder might take the part of filler due to the small dimension of the units and fill up the holes. The formation of C-S-H from the reaction of higher amounts of C_3_S and water resulted in early age strength.

The maximum compressive strength was for samples with 5% SSW at 90 days which could be due to a higher amount of C_2_S in the sample. It can be seen from Figure 6 that the compressive strengths of samples containing 10% SSW powder were increased by 7.4%, 13.3%, 11.7% at 7, 28, and 90 days, respectively, compared to the control samples. These findings indicate that as the SSW powder ratio increased from 5% to 10%, the compressive strengths decreased due to the reduced amount of the replacement of cement. The compressive strength of the specimens of late age was depleted by the addition of more than 10% SSW, possibly due to a smaller amount of C_2_S in the concrete related to the high substitute part of cement.

### 3.3. Tensile Strength of Samples

**Figure 7:**
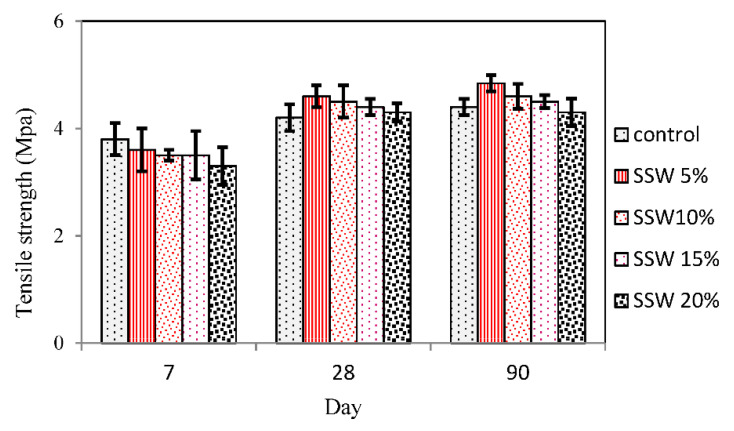
Tensile strengths of samples with SSW powder.

A tensile strength test was performed according to ASTM C 496 [30] for control and samples containing 5, 10, 15, and 20% SSW. Figure 7 illustrates the tensile strength of samples at 7, 28, and 90 days of age. It was observed that the replacement of SSW powder lowered the tensile strength at an early age. Figure 7 demonstrates that 5% SSW decreased the 7th day tensile strength by up to 5.2%. In Figure 7, a slight reduction in tensile strength of samples containing 10, 15 and 20% SSW was observed. The decreased rate in tensile strength at an early age can be due to the creation of weak C–S–H [31,32]. Moreover, as can be seen from Figure 7, the replacement of SSW powder with cement increased the tensile strength at later ages. The tensile strength was increased by 10.46%, 7.1%, 4.7%, and 2.4% due to the greater availability of C_2_S when cement was substituted with 5%, 10%, 15%, and 20% SSW, respectively. The tensile strength declined when 20% SSW was used compared with 5% SSW. In the 5% SSW samples, the reactions started after 7 days and continued until 90 days. Therefore, the tensile strength of samples with 5% SSW was improved.

### 3.4. SEM of Samples

**Figure 8:**
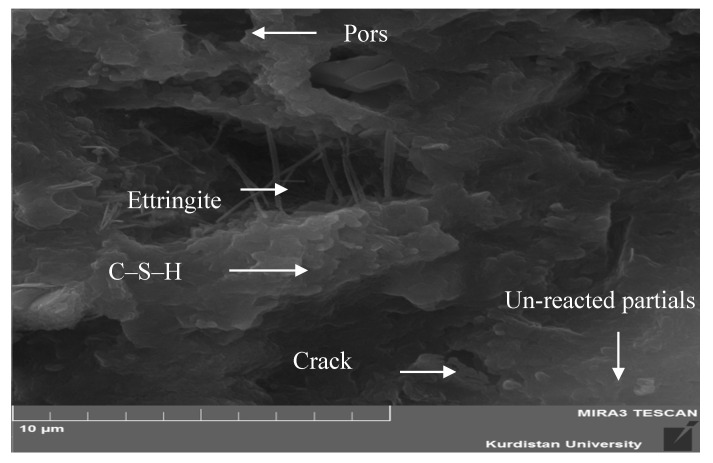
SEM image of control sample.

SEM image of the control and 5% SSW samples were carried out. Figure 8 illustrates the microstructure of control samples at 28 days. A formation of Ettringite, pores, and cracks were observed in control samples. Unreacted partials, C-H, and C-S-H formation were also observed in the control samples. The available spaces in the cement paste, the heat of hydration process, and the amount of impurities of the cement affect the hexagonal crystals formed in the C-H [31]. The role of C-H is restricted because of low Van der Waals forces affecting concrete strength. This is due to its low surface area [16].

**Figure 9:**
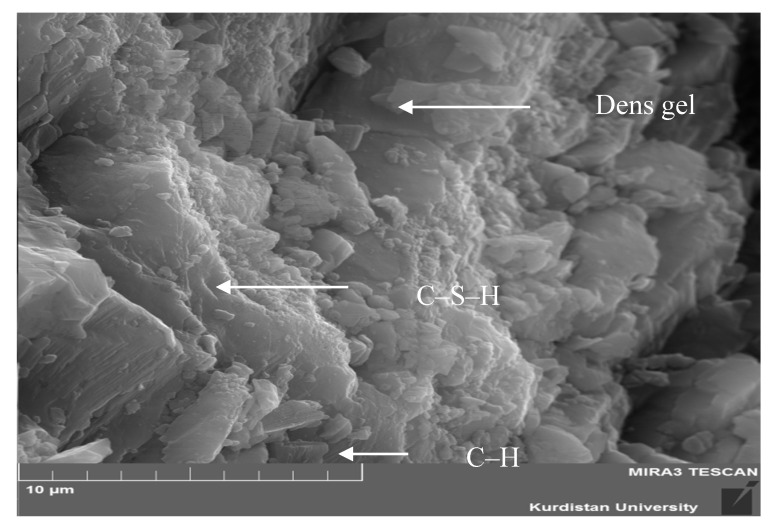
SEM image of sample containing 5% SSW.

A progressive process of hydration and the creation of C-S-H clusters were observed in control samples. The C-S-H formation possesses a layered structure with high surface area [32]. The shape of the C-S-H formation differs in nature from weak fiber crystals to dense matrices [33]. The SEM images of samples containing 5% SSW at 28 days of hydration are shown in Figure 9. As observed, the addition of SSW powder changed the morphology with no visible pores. Figure 8 and Figure 9 illustrate the visible changes in the chemical composition. The Microstructure of Figure 9 with 5% SSW was denser than Figure 8. In Figure 9, more C-S-H formation can be seen throughout the matrix. High amounts of Si in SSW powder might raise the amount of C-S-H compared to the control sample.

### 3.5. EDX Analysis of Samples

**Figure 10:**
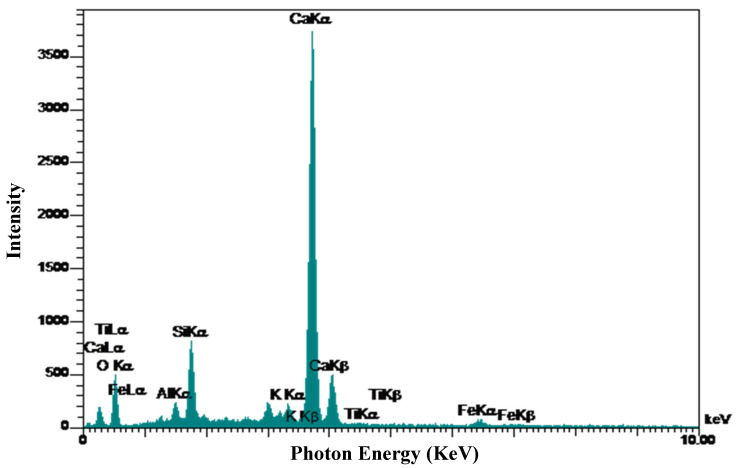
Energy-dispersive X-ray microanalysis (EDX) results of control specimen.

**Figure 11:**
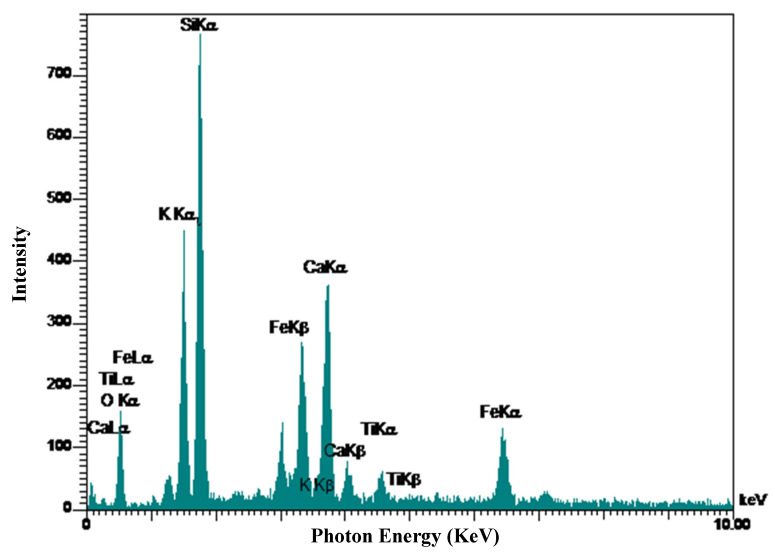
EDX results of specimen containing 5% SSW.

Energy-dispersive X-ray microanalysis (EDX) was conducted. Figure 10 shows EDX analysis results of the control sample. As shown in Figure 10, the control sample contains a higher percentage of Ca and the major elements present are Si, O, Fe, and Al. The recognized elements in the EDX analysis results match with the chemical components of cement shown in Table 1. Due to the amounts of Al component, the control sample seen by EDX analysis in Figure 10 has the potential for Ettringite formation, as already demonstrated in Figure 8. Ti was found in the control sample which could have originated in the aggregates. The EDX analysis results of samples containing 5% SSW is presented in Figure 11. The Ca and the Si ratios are 36.50% and 79.3%, respectively. The high amount of silica in EDX analysis of samples with 5% SSW and a very low content of other elements might show that SSW powder was as a result of the C-S-H formation instead of other weak formations.

### 3.6. FTIR Analysis of Samples

**Figure 12:**
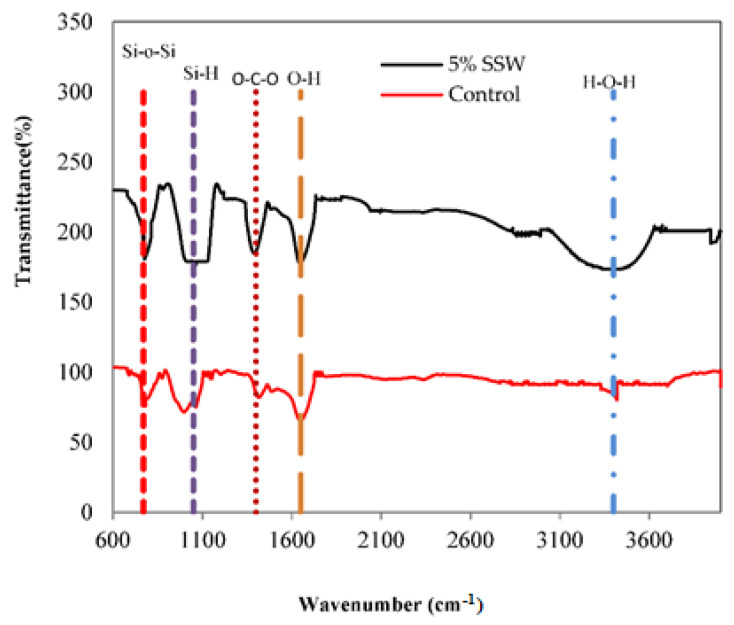
FTIR spectra of control and concrete with 5% SSW.

The FTIR spectra of control and sample with 5% SSW were distinguished in Figure 12. The changes in the locations of the peaks in the FTIR spectra of the concrete with 5% SSW reveal that the C-S-H formation transformed over time. Figure 12 presents the C-S-H chains through bands characteristics spectra of Si-O-Si, Si-OH, O-C-O, O-H, and H-O-H. The bands were annotated in Figure 12. The changes in H-O-H bending stretch at 3400 cm^−1^ were not more distinctive in the FTIR spectra. The bands at 1400 and 1650 cm^−1^ were because of the asymmetric and symmetric Si–O stretching vibrations. The stretching vibrations band at 1120 cm^−1^ is assigned to Si-OH as reported in the previous study [34]. The strong bands at 770 cm^−1^ correspond to the Si-O-Si band that can prove a greater amount of C-S-H formation [35]. It can also be seen that the addition 5% of SSW to control increased the band of Si-O-Si [24]. The reason can be justified by the reaction of 5% SSW with cement in which C-H is used and as a result, more C-S-H is created, which finally improves the compressive strength of samples [36,37].

### 3.7. XRD Graphs of Samples

**Figure 13:**
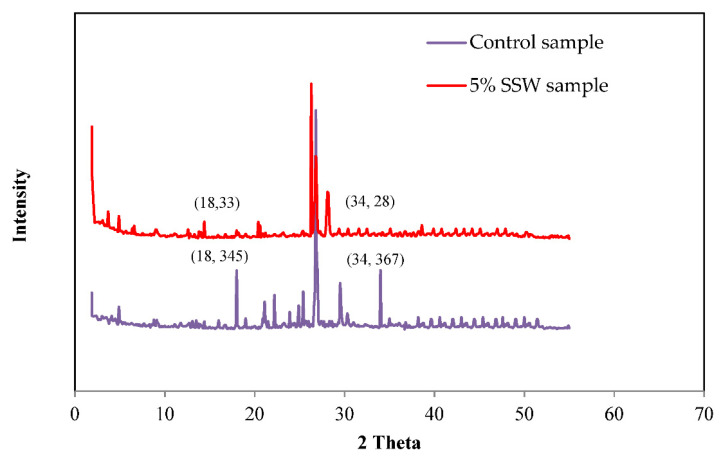
XRD pattern of the control and the 5% SSW samples at 28 days.

In Figure 13, the intensity peaks of the quartz phase were traced in the recorded XRD graphs of the control and 5% SSW sample at 28 days of the curing age. In the mentioned XRD graph, C-H crystal was noted at 2 Theta of 18° and 34° [22,25,38]. The C-H intensity of the control sample was 345 at 18° and 367 at 34° (2 Theta). Furthermore, Figure 13 shows the XRD graph of 5% SSW sample at 28 days in which the C-H intensity of 5% SSW sample was 33 at 18° and 28 at 34° (2 Theta). This may due to the reaction of SiO_2_ with the C-H from the subsequent hydration of cement, which was reported in previous work [39,40]. The decreases of C-H intensity for 5% SSW sample were 90% and 92% at 18° and 34° (2 Theta), respectively.

The crystals of C-H were utilized and reactions were generated through the usage of 5% SSW. Thus, the XRD graphs illustrate that 5% of SSW led to a rise in the C-S-H formation, indicating that the compressive strength, SEM, EDX, and FTIR results from 5% SSW sample are compatible with the XRD graphs. It can be observed that the replacement of cement with 5% SSW depleted the peaks compared with the control sample because of the formation of a new amorphous material [38,41,42].

## 4. Conclusions

For the first time, the present paper studies the effects of silica stone waste on the properties of concrete. Cement was replaced with 5%, 10%, 15%, and 20% of the silica stone waste. Slump test, compression, tensile strength test, SEM, EDX FTIR, and XRD were performed to trace the microstructure and chemical composition of the specimens that were undertaken to assess the mechanical properties and microstructure of samples. Results indicate that the Slump of samples containing the SSW powder increase could be due to decreased water demand that cannot be absorbed by silica stone waste. Results from the test showed that the addition of 5% silica stone waste as cement replacement increased the mechanical properties in all ages.

Replacement of 5% silica stone waste augmented the compressive strength by 18.8% at 28 days due to secondary C-S-H formation and filler effect of their particles. The tensile strength with 5% SSW was improved by up to 10.46%. It could be concluded that secondary C-S-H formation was observed when silica stone waste was added. Furthermore, the high amount of silica in EDX analysis of samples with 5% SSW and a very low content of other elements might show that SSW powder was as a result of the C-S-H formation instead of other weak formations such as C-H. Furthermore, according to FTIR and XRD graphs, C-S-H formation was found in concrete treated by using 5% of SSW. It is worth mentioning that integration of the other mechanical properties, for instance, the strengths and elastic evaluations would remain as the future research direction.

## Figures and Tables

**Table 1:** Chemical and physical components of cement and SSW.

Chemical Components, %	Cement	SSW
SiO_2_	22.2	96.24
Al_2_O_3_	3.89	1.13
Fe_2_O_3_	3.64	0.99
CaO	62.96	0.83
MgO	1.59	0.19
Other	5.7	0.62
**Physical Components**		
Specific gravity, g/cm^3^	3.0	2.65
Average size, Micron	13.7	8

**Table 2:** Mix design of concrete.

Mix Design	W/B	Cement kg/m^3^	Water kg/m^3^	SSW kg/m^3^	Coarse Aggregate kg/m^3^	Fine Aggregate kg/m^3^
Control	0.41	445	270	0	1000	746
SSW _5%_	0.41	422.75	270	22.25	1000	746
SSW _10%_	0.41	400.5	270	44.5	1000	746
SSW _15%_	0.41	375.25	270	66.75	1000	746
SSW _20%_	0.41	356	270	89	1000	746
